# Measles and Mumps in Combination

**Published:** 1833

**Authors:** 


### III. Measles and Mumps in Combination.

Dr. A. W. Bowen, of the town of Russia, in the state of New York, has lately met with Mumps and Measles at the same time, in a number of patients, and has politely favored us with some brief notices of the combination. In 1832, from April to June, these two diseases prevailed extensively in the region where he resides.

"As a general remark, the Measles were mild, while on the contrary, the Mumps were almost invariably severe, and frequently attended with metastasis to the testicles. Some cases of the latter were attended with enormous swelling and high inflammatory excitement, requiring the lancet and other antiphlogistic remedies. Although a great many adult females were attacked with Mumps, and some while laboring under the Measles, yet I saw no case of translation to the breasts—nor heard of any. As instances of the close intimacy of Measles and Mumps, two cases, recorded at the time, are given below —they are only fair samples of a great manysimilar ones which occurred in the same district.

Miss H. Moon, Æt. 17, was attacked with Mumps May 3d, 1832—glands enlarged considerably—jaws seperated with the utmost pain and difficulty, attended with considerable inflammatory action. She was put on light diet and purged with sulphate of Magnesia. On the third day from her attack, she broke out finely with Measles. Her recovery was as rapid and as perfect as is usual in cases of either of these diseases.

On the 10th of the same month, her sister Susan, aged 15, broke out with the Measles, and the next day, the parotid gland of the left side, swelled with all the characteristics of Mumps; she recovered rapidly with the ordinary antiphlogistic treatment—without the lancet.

Of the cases of Measles probably nine out of ten required no medical treatment; there were, however, some few exceptions, which required all the skill and ingenuity of the science to combat. Of the the cases of Mumps in adult males, probably more than half were attended with translation, and required the most active remedies. As a local application to the scrotum, none appeared to afford so much relief, as wheat bran wet with a solution of acetate of lead in vinegar —or with vinegar alone—applied by means of a bandage around the hips, in such a manner as to support the testicle—as its own weight without such support, was of itself tormenting."

Measles and Mumps are dependent on different remote causes of a specific character; and the facts here narrated, prove that they may simultaneously develope their pathological effects in the same patient, one, however, affecting the skin and mucous membrane—the other the parotid glands.

